# Curved and Annular Diaphragm Coupled Piezoelectric Micromachined Ultrasonic Transducers for High Transmit Biomedical Applications

**DOI:** 10.3390/s24092714

**Published:** 2024-04-24

**Authors:** Yun Zhang, Tong Jin, Zijie Zhao, Chenfang Yan, Xinchao Lu, Hang Gao, Chengjun Huang

**Affiliations:** 1 Institute of Microelectronics of the Chinese Academy of Sciences, Beijing 100029, China; zhangyun@ime.ac.cn (Y.Z.); jintong@ime.ac.cn (T.J.); yanchenfang@ime.ac.cn (C.Y.); luxinchao@ime.ac.cn (X.L.); huangchengjun@ime.ac.cn (C.H.); 2 University of Chinese Academy of Sciences, Beijing 100049, China

**Keywords:** piezoelectric micromachined ultrasonic transducers (pMUTs), coupled structure, curved and annular diaphragm, high transmit efficiency, analytical equivalent circuit model (EQC)

## Abstract

In this paper, we present a novel three-dimensional (3D) coupled configuration of piezoelectric micromachined ultrasound transducers (pMUTs) by combing a curved and an annular diaphragm for transmit performance optimization in biomedical applications. An analytical equivalent circuit model (EQC) is developed with varied excitation methods to incorporate the acoustic–structure coupling of the curved and annular diaphragm-coupled pMUTs (CAC-pMUTs). The model-derived results align well with the reference simulated by the finite element method (FEM). Using this EQC model, we optimize the key design parameters of the CAC-pMUTs in order to improve the output sound pressure, including the width of the annular membrane, the thickness of the passive layer, and the phase difference of the driving voltage. In the anti-phase mode, the designed CAC-pMUTs demonstrate a transmit efficiency 285 times higher than that of single annular pMUTs. This substantial improvement underscores the potential of CAC-pMUTs for large array applications.

## 1. Introduction

In the past decades, ultrasonic transducers have gained increasing popularity in applications such as medical imaging [1,2,3], invasive or non-invasive therapy [4,5], biomedical particle manipulation [6,7,8], gesture recognition [9], and neural stimulation [10]. The high transmit performance of the transducers is key for these applications to achieve enhanced pressure output with good signal-to-noise contrast (SNC). Conventional piezoelectric ceramic ultrasonic transducers, operating in the thickness mode [11], are generally processed by hand cutting. Firstly, there is a mismatch in the acoustic impedance between the transducer and the loading medium. Although a matching layer or ultrasonic coupling agent is usually embedded in between, the loss of acoustic energy and the extra fabrication process of the matching layer still cannot be neglected. In addition, conventional hand-cutting methods face challenges in producing high fill-factor transducer arrays with patterned layouts [12]. In such arrays, optimizing the interference of individual elements is crucial for maximizing the output. To address these issues, micromechanical ultrasonic transducers (MUTs) based on microelectromechanical systems (MEMS) technology has been developed, aiming to achieve low-cost and large-depth ultrasound detection [13]. MUTs can be categorized into capacitive micromechanical ultrasound transducers (cMUTs) and piezoelectric micromechanical ultrasonic transducers (pMUTs). cMUTs, utilizing electrostatic forces to convert electrical energy into acoustic energy, have been demonstrated to have good performances such as high electromechanical coupling coefficients and large bandwidths. Nevertheless, the large DC bias needed for actuation induces the device at a high risk of damage, and limits the applications of cMUTs in biomedical situations [11]. Additionally, the small cavity gap required by the high receive sensitivity limits the vibration amplitude of the membrane and represents a challenge for transmit efficiency [14]. In contrast, pMUTs have no limitation on DC bias or cavity gap due to their use of thin-film piezoelectric effects to achieve ultrasound transduction. Furthermore, pMUTs also have the advantages of a lower electrical impedance [15], linear drive response, and large vertical deflection [12]. pMUTs have always been an important research direction for micro ultrasound experts [16]. However, pMUTs normally suffer from low electromechanical coupling coefficients [14], limiting the efficiency of converting electrical energy into mechanical energy. Additionally, acoustic coupling remains less optimized and therefore prevents the realization of high-pressure outputs [17].

Ultrasonic transmit performance must be optimized to reach a large penetration depth and a high SNC [18,19,20]. To achieve this, various studies have been reported, which can be mainly divided into two categories: improving the electromechanical coupling coefficient of the microstructures or optimizing their acoustic coupling characteristics.

Based on circular diaphragms, Amar et al. [21] developed a lumped parameter model to optimize the design of PZT pMUTs and investigate the effect of electrode coverage and substrate thickness on the performance of pMUTs. Furthermore, Sammoura et al. [22] constructed an equivalent circuit (EQC) model of a pMUT with multiple electrodes, which significantly increased the electromechanical coupling coefficient by 211% over the state-of-the-art single-electrode design. Akhbari et al. [19] designed a two-electrode bimorph pMUT for air- and liquid-coupled applications, increasing the electromechanical coupling efficiency by a factor of 4 compared to devices with similar structures and frequencies. Ring-structured pMUTs also presented their capability in enhancing the output pressure of the device [23]. A ring-structured pMUT designed by Eovino et al. [24] demonstrated 11.8 times higher sound pressure and 9 times higher directionality. When considering 3D vibration diaphragms, many studies have investigated the significance of the dome-shaped or spherical structures on their electromechanical coupling efficiency. In such curved structures, when applying an electric field, the initial in-plane strain leads to a larger vertical deformation because of clamping and curvature around the periphery. In addition, eliminating the rigid passive contributor in the vibration layers also contributes to more efficient energy transfer into the mechanical domain [14,15,25]. Morris et al. [26] introduced a static pressure bias to the diaphragm to form an initial curvature, converting the compressive deformation of the diaphragm surface into the amplified pressure output. Feng et al. [27] proposed a microfabrication technique for dome-shaped transducers using ZnO as the piezoelectric film. The radius and curvature of the dome-shaped film is precisely controlled, resulting in 200 mV pulse-echo signals at 215 MHz. Akhbari et al. [12] designed curved pMUTs with a pressure level that is 50 times higher than that of planar pMUTs.

In contrast, fewer studies have focused on optimizing the acoustic coupling of pMUTs, primarily due to the complex nature of their acoustic interactions, which can alter the acoustic loading of each structure. Shelton et al. [28] added impedance-matched resonator tubes below the MUT to increase the acoustic coupling of the small-sized device, leading to a 350% increase in the transducer’s sound pressure level (SPL). Xu et al. [29] developed a Helmholtz resonant (HR) cavity-integrated pMUT, achieving a pressure increase of up to 163% compared with that of the circular counterpart because of the liquid amplification effect. Feng et al. [30] proposed a pMUT integrated with a volume-tunable Helmholtz resonator, realizing a 200% sound pressure level (SPL) increase and two-fold bandwidth enhancement in the experiments. Cheng et al. [31] enhanced the receive sensitivity of the pMUT by applying a DC bias during operation, improving sensitivity by a factor of 2.5 using a 15 V DC bias. Thereafter, Xu et al. [17] proposed a pMUT structure combing annular and circular diaphragms, resulting in an output pressure increase of 155% compared with that generated by the circular reference. Given the high-pressure output of curved structures, it is crucial to investigate the acoustic coupling mechanisms inherent in the three-dimensional diaphragms of pMUTs.

In this work, we couple a curved structure and an annular structure concentrically as a novel pMUT configuration, referred to as a curved and annular coupled pMUT (CAC-pMUT). Given the enhanced electromechanical properties of the curved structure and the coupling acoustic effects, the CAC-pMUTs can achieve any assigned resonance frequency with high output performance. In order to optimize the design, we developed an EQC model by introducing the coupling acoustic impedances, which allows for a quantitative characterization of key coupling effects both in mechanical domains and acoustic domains.

The model-derived results are validated by finite element method (FEM) simulations. Based on the EQC model, we studied the key impactors of the transmit pressure, including the width of the annular membrane, the thickness of the passive layer, and the phase difference of the driving voltage. A comparison study shows that CAC-pMUTs driven in an anti-phase manner present better output pressure performance than single annular pMUTs.

## 2. Equivalent Circuit Model for CAC-pMUT

### 2.1. Design of CAC-pMUT

**Figure 1:**
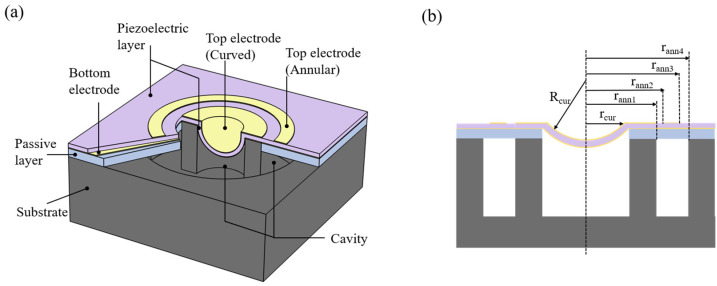
(**a**) Schematic of CAC-pMUT coupled with spherical and annular structures. (**b**) Side view of a coupled pMUT structure.

As shown in Figure 1a, in this coupled pMUT, the curved structure is located in the middle, and the annular pMUT is coaxially located at the periphery area. The annular structure is fabricated on a substrate and contains both active and passive layers. In contrast, a curved counterpart eliminates a passive layer due to the existence of curvature. Across the annular and the curved structures, the piezoelectric layer sandwiched by top electrodes and bottom electrodes shows consistency in thickness and actively vibrates. In theory, the coupling effects between the curved and the annular structures mainly include the mechanical crosstalk and the acoustic crosstalk. In this design, however, the top electrodes of the annular and curved structures are discrete and can be driven separately. Thus, the EQC model of the CAC-pMUT can be simplified by computing mechanically independent individual annular and individual curved plates, where mechanical crosstalk is neglected and only interactions through the acoustic medium are considered.

Acting as the vibration layer, the active layer is an AlN piezoelectric thin film, while the passive layer of the annular structure is made of Si. Given that the non-zero piezoelectric moment of the annular part requires the falling of the neutral axis in the passive layer [20], the thickness of the passive layer must be larger than that of the active layer. To ensure the strength of the cavity etching [17], a gap of approximately 35 μm is placed between the coaxial curved and annular films.

### 2.2. Equivalent Circuit Model

The equivalent circuit model of the CAC-pMUTs is developed in three steps, including theoretically modelling a single curved pMUT, analytically modelling a single annular pMUT, and thereafter incorporating the acoustic mutual impedance of the CAC-pMUT.

**Figure 2:**
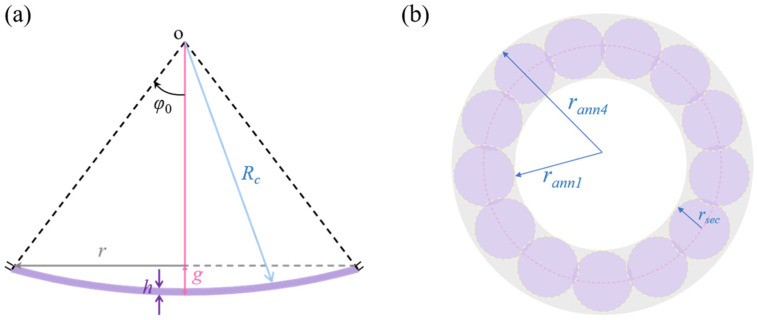
(**a**) Two-dimensional schematic of a clamped curved pMUT. (**b**) Schematic of an annular pMUT which is divided into approximations by a number of circular pistons.

In Figure 2a, there are several important parameters for curved devices, namely, the radius of curvature *R_c_,* the nominal radius *r*, and the film thickness *h*. The azimuthal angle *φ*_0_ and the distance from transducer center to the baffle plane ‘*g*’ can be expressed as [12,25]

(1) $$\varphi_{0}={sin}^{-1}\left[\frac{r}{R_{c}}\right]$$

(2) $$g=R_{c}\left(1-\cos\varphi_{0}\right)$$

The equivalent circuit model for a curved pMUT in transmission mode is first investigated. In transmission mode, the curved pMUT generates a volumetric displacement under the input excitation voltage. The volumetric displacement equation can be calculated as [12,25]

(3) $$\begin{array}{ll} Wvol & =2\pi {R_{c}}^{2}[\left(1-cos\varphi_{0}\right)+A_{1}^{\prime }H\left(l_{1}\right)+\\ & A_{2}^{\prime }H\left(l_{2}\right)+A_{3}^{\prime }H\left(l_{3}\right)]\left[p+\frac{2Y_{0}d_{31}V}{R_{c}(1-v)}\right]b(\omega ) \end{array}$$

(4) $$H\left(l_{x}\right)=\frac{\left[P_{l_{x}-1}\left(\cos\varphi_{0}\right)-P_{l_{x}+1}\left(\cos\varphi_{0}\right)\right]}{\left[1+2l_{x}\right]}$$

(5) $$l_{x}={\left(\lambda_{x}+\frac{1}{4}\right)}^{0.5}-\frac{1}{2}x=1,2,3$$

where *Y*_0_ is the Young’s modulus, *d*_31_ is the piezoelectric coefficient, *v* is the Poisson’s ration of the piezoelectric material, *p* is the incident pressure, *V* is the input voltage, and *λ_x_* are the solutions of the characteristic equation [25]:

(6) $$\lambda^{3}-\left[4+\left(1-v^{2}\right)\Omega^{2}\right]\lambda^{2}+12\left(1-v^{2}\right){\left(\frac{R_{c}}{h}\right)}^{2}\left(1-\Omega^{2}\right)\lambda -\;12\left(1-v^{2}\right){\left(\frac{R_{c}}{h}\right)}^{2}\left[2+\left(1+3v\right)\Omega^{2}+\left(v^{2}-1\right)\Omega^{4}\right]=0$$

(7) $$\Omega^{2}=\frac{\rho \omega^{2}{R_{c}}^{2}}{Y_{0}}$$

where *ω* is the angular frequency, *ρ* is the density of the piezoelectric layer, and *h* is the thickness. The value of *A_x_*′ is determined by the operating frequency and the clamped boundary conditions. *b*(*ω*) is the frequency dependent [25]:

(8) $$b\left(\omega \right)=\frac{\left(1-v\right)+\left(1-v^{2}\right)\Omega^{2}}{\left[2+\left(1+3v\right)\Omega^{2}+\left(v^{2}-1\right)\Omega^{4}\right]}\left(\frac{{R_{c}}^{2}}{hY_{0}}\right)$$

*P_i_* is the Legendre function of the first kind of order *i*. The calculation of Legendre functions of a complex degree can be completed by using the Mehler integral of the associated Legendre function [32]:

(9) $${P_{n}}^{m}\left(\cos\varphi \right)={\left(\frac{2}{\pi }\right)}^{0.5}\frac{{sin}^{m}\varphi }{\Gamma \left(0.5-m\right)}\int_{0}^{\varphi }\frac{\cos\left(n+0.5\right)x}{{(\cos x-\cos\varphi )}^{m+0.5}}dx$$

Thus, the volumetric displacement can be rewritten as a function of the input voltage and the incident sound pressure:

(10) $$Wvol=Y_{m}p+b_{t}V$$

(11) $$\begin{array}{c} {Y_{m}}_{cur}=2\pi {R_{c}}^{2}[\left(1-cos\varphi_{0}\right)+A_{1}^{\prime }H\left(l_{1}\right)+\\ A_{2}^{\prime }H\left(l_{2}\right)+A_{3}^{\prime }H\left(l_{3}\right)]b(\omega ) \end{array}$$

(12) $$\begin{array}{ll} {b_{t}}_{cur} & =\left[\frac{4\pi Y_{0}d_{31}R_{c}}{1-v}\right][\left(1-cos\varphi_{0}\right)+A_{1}^{\prime }H\left(l_{1}\right)+\\ & A_{2}^{\prime }H\left(l_{2}\right)+A_{3}^{\prime }H\left(l_{3}\right)] \end{array}$$

where *Y_m_* is the mechanical admittance, and *b_t_* is the transduction coefficient.

As a result, the mechanical impedance and electromechanical conversion efficiency in the EQC model of the curved pMUT can be calculated as

(13) $$Z_{m\_cur}=\frac{1}{j\omega Y_{m_{cur}}}$$

(14) $$\eta_{{\_}_{cur}}=\frac{b_{t_{cur}}}{Y_{m_{cur}}}$$

Radiation impedance is another essential parameter in the EQC model of a curved pMUT device. When the distance from transducer center to the baffled plane *g* of a curved pMUT is much smaller than the nominal radius r, the curved pMUT can be approximated as a flat pMUT for the calculation of the acoustic impedance. The acoustic impedance of the flat piston pMUT is calculated as [33]

(15) $$Z_{a_{cur}}=k_{c1}\rho_{m}c\pi {r_{eff}}^{2}\left\{\left[1-\frac{J_{1}\left(2kr_{eff}\right)}{kr_{eff}}\right]+j\left[\frac{H_{1}\left(2kr_{eff}\right)}{kr_{eff}}\right]\right\}$$

where *ρ_m_* is the density of the medium, *c* is sound speed in the corresponding acoustic medium, *k* is the wave number, and *r_eff_* is the effective radius of the clamped flat or curved pMUT. *J*_1_ is the first order of the first kind Bessel function and *H*_1_ is the first kind of the Struve function. *k_c_*_1_ is a correction factor [12], and for the curved pMUT, it is shown that *k_c_*_1_ should be taken as 0.82.

The next step is to model the EQC of the annular membrane. As shown in Figure 2b, one annular pMUT can vibrate similarly as a bundle of circular pistons. Based on this assumption, we can characterize the mechanical and acoustic impedances of the proposed annular pMUT by calculating the counterparts of an array of circular pistons. The number of small circular pistons, *N*, can be calibrated by modelling the vibrations of a single complete annular piston diaphragm [17,34]. The radius of the small circular piston *r_sec_* can be calculated as

(16) rsec=πrann42−rann12N

The volumetric displacement resulting from the vibration of the annular pMUT can also be expressed as a function of the incident pressure and the excitation voltage as shown in Equation (10), where the mechanical admittance can be expressed as the volumetric displacement generated by per unit of incident sound pressure, and the electromechanical transduction ratio can be expressed as the volumetric displacement produced per unit voltage [34]:

(17) $$Y_{m_{ann}}={\frac{2\pi }{p}\left.\left[\int_{r1}^{r2}W_{1}\left(r\right)rdr+\int_{r2}^{r3}W_{2}\left(r\right)rdr+\int_{r3}^{r4}W_{3}\left(r\right)dr\right]\right|}_{V=0}$$

(18) $$b_{t_{ann}}={\frac{2\pi }{V}\left.\left[\int_{r1}^{r2}W_{1}\left(r\right)rdr+\int_{r2}^{r3}W_{2}\left(r\right)rdr+\int_{r3}^{r4}W_{3}\left(r\right)dr\right]\right|}_{p=0}$$

where *r*_1_ is the inner radius of the annular structure, *r*_4_ is the outer radius, *r*_2_ is the inner radius of the top electrode, and *r*_3_ is the outer radius of the top electrode. In the modelling, the annular diaphragms are divided into three segments by the width of the electrode, including regions under the electrode and outside the electrode. *W*_1_(*r*), *W*_2_(*r*), and *W*_3_(*r*) in (17) and (18) are the shape functions of each segment, and the combined term in parentheses indicates the volumetric displacement of the annular membrane. Therefore, the mechanical impedance and electromechanical transduction ratio of the annular pMUT can be expressed as

(19) $$Z_{m\_ann}=\frac{1}{j\omega Y_{m_{ann}}}$$

(20) $$\eta_{\_ann}=\frac{b_{t_{ann}}}{Y_{m_{ann}}}$$

Accordingly, the mechanical impedance of each sector is

(21) $$Z_{m\_sec}=\frac{Z_{m\_ann}}{N}$$

(22) $$\eta_{\_sec}=\eta_{\_ann}$$

The acoustic impedance of the annular pMUT can be calculated as the reference of the small circular piston array, including the self-radiation impedance and the mutual radiation impedance. The self-radiation impedance acts as the damping effect on itself when it vibrates, whereas the mutual radiation impedance refers to the effect between different elements through the acoustic medium. Firstly, the self-radiation impedance is calculated as

(23) $$Z_{a\_sec}=\rho_{m}c\pi {r_{sec}}^{2}\left\{\left[1-\frac{J_{1}\left(2kr_{sec}\right)}{kr_{sec}}\right]+j\left[\frac{H_{1}\left(2kr_{sec}\right)}{kr_{sec}}\right]\right\}$$

The mutual radiation impedance is a function of the pitch of sectors *d* and the wavenumber *k*, and the formula are as follows [35]:

(24) $$Z_{mn}=\rho_{m}c\pi {r_{sec}}^{2}A\left(kr_{sec}\right)\frac{\sin kd+j\cos kd}{kd}$$

The function ‘A’ is found by curve fitting a polynomial of the 10th order [35].

**Figure 3:**
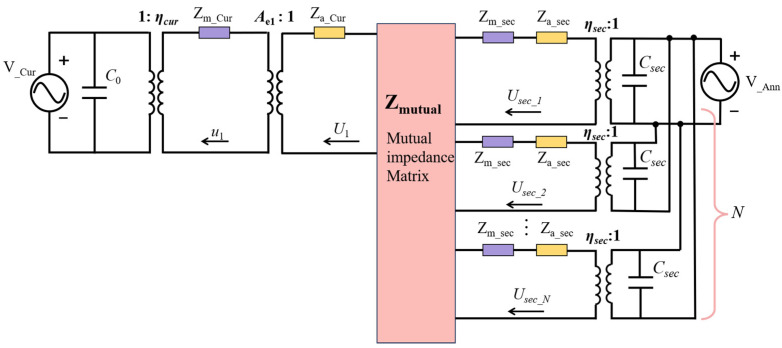
EQC model of the CAC-pMUT by dividing the annular into *N* parts, each part approximating a circular piston.

In Figure 3, the EQC model of the CAC-pMUT presents as the concentrically coupled structure of the curved pMUT and the annular pMUT. Given that acoustic interactions dominate in the proposed design, we therefore characterize the coupling effects of the curved membrane and the equivalent circular piston array as the mutual radiation impedance in the acoustic domain. In the calculation of the self-radiation impedance of the curved pMUT, since the *g* of the curved membrane is much smaller than the nominal radius, the curved pMUT is also approximated as a flat transducer. The mutual radiation impedance of two pMUTs of different radii can be expressed as [36]

(25) $$\begin{array}{ll} Z_{ij}= & k_{c2}\times 2\rho_{m}c\pi {r_{eff}}^{2}\times \\ & \sum_{p=0}^{\infty }\{\frac{1}{\pi^{0.5}}1\Gamma \left(p+\frac{1}{2}\right){\left(\frac{r_{eff}}{d_{ij}}\right)}^{p}[{\left(\frac{\pi }{2kd_{ij}}\right)}^{0.5}H_{p+0.5}^{\left(2\right)}\left(kd_{ij}\right)]\\ & \times \sum_{n=0}^{p}{\left(\frac{r_{sec}}{r_{eff}}\right)}^{n+1}[\frac{J_{p-n+1}\left(kr_{eff}\right)J_{n+1}\left(kr_{sec}\right)}{n!\left(p-n\right)!}]\}\; \end{array}$$

where *Γ* is the gamma function and *H*^(2)^*_x_* is the spherical Hankel function of the second kind [17]. *d_ij_* is the distance between two centers of the curved structure and the circular sectors. *k_c_*_2_, is a correction factor and turns out to be about 0.91 in this study.

### 2.3. Validation of Equivalent Circuit Model

**Figure 4:**
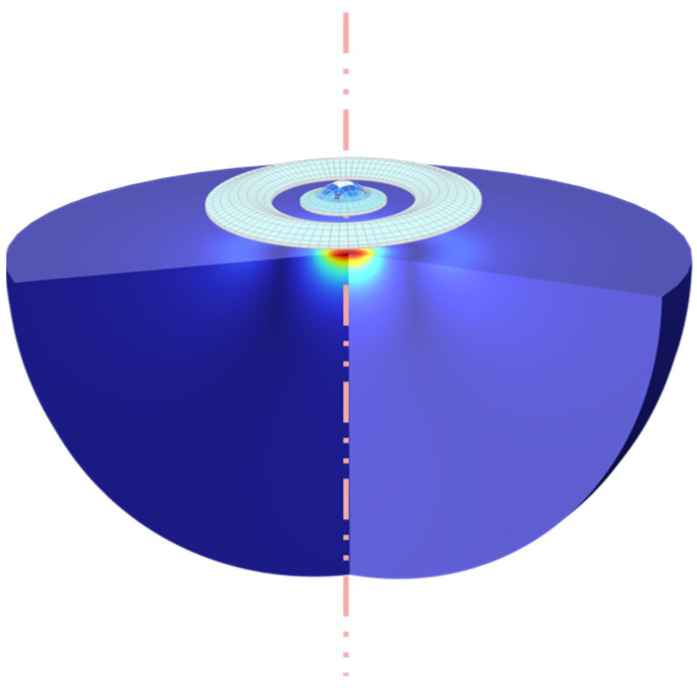
Schematic of a COMSOL model for a CAC-pMUT.

In order to validate the EQC model, COMSOL Multiphysics (COMSOL 6.0), is used as the FEM tool to exam the dynamic response characteristics in water (*ρ_m_* = 1000 kg/m^3^, *c* = 1500 m/s), including the curved pMUT, the annular pMUT, and their combination CAC-pMUT. Figure 4 exemplifies a schematic of a COMSOL model for a CAC-pMUT. In the finite element model, the outer boundary of the curved membrane as well as the inner and outer boundaries of the annular diaphragm are clamped, and a radiation boundary condition for a spherical wave is added in the acoustic domain for allowing minimum reflections. We have used a 2D model for quick validation and a 3D model for more informative explorations such as viewing the vibration modes. In the mesh, the mechanical and acoustic domains are dissected with free triangular in case of 2D models and free tetrahedral in case of 3D models. In these cases, the maximum mesh size in the acoustic domain is defined as one-sixth of the wavelength or less.

The detailed geometric and material parameters of the pMUTs are summarized in Table 1 and Table 2, where the top electrode coverages in the curved structure and in the annular pMUT are 100% and 55%, respectively.

According to Kirchhoff’s voltage law (KVL), the velocity averaged over the effective area Scur of the curved pMUT can be presented as

(26) $$u_{ave_{cur}}=\frac{V_{cur}\times \eta_{{\_}_{cur}}}{Z_{m_{cur}}+\frac{Z_{a_{cur}}}{{S_{cur}}^{2}}}\;$$

where the *V_cur_* is the excitation voltage applied to the top electrode and is set to 1 V in amplitude.

Given that the annular pMUT can be divided into a number of sectors, its average velocity is equivalent to the average velocity of the circular pistons in one array. The volumetric velocity of the circular piston in array [12] is

(27) $${v_{sec}}_{N\times 1}={\left\{I_{N\times N}{Z_{m\_sec}}_{N\times N}+{Z_{S}}_{N\times N}\right\}}^{-1}diag\left({\eta_{\_sec}}_{N\times 1}{V_{ann}}_{1\times N}\right)$$

where *V_ann_* is the excitation voltage of the annular pMUT and is set as 1 V in amplitude. *Z_s_* is a *N × N* acoustic impedance matrix of the circular piston array, as

(28) $$Z_{S}=\left[\begin{array}{cccc} Z_{a\_sec} & Z_{11} & \cdots & Z_{1N}\\ \; & Z_{a\_sec} & \cdots & Z_{2N}\\ \; & \; & \ddots & \vdots \\ \; & \; & \; & Z_{a\_sec} \end{array}\right]$$

and the average velocity of the sector is

(29) $${u_{ave}}_{ann}={u_{ave}}_{sec}=\frac{v_{sec}}{S_{sec}}$$

*v_sec_* is the volumetric velocity of any sector as the value is the same for each sector. *S_sec_* is the effective area of the circular sector.

**Figure 5:**
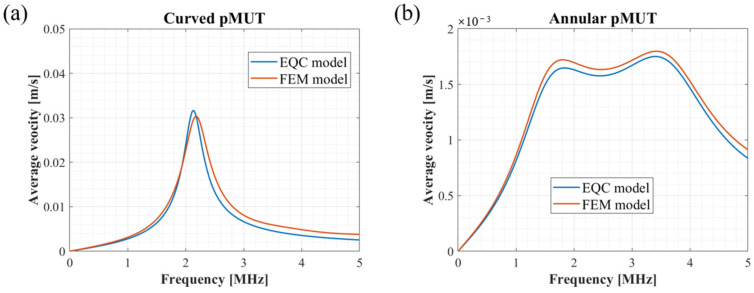
Dynamic response of a single curved pMUT (**a**) and a single annular pMUT (**b**).

Figure 5 shows the dynamic response of the single curved pMUT and the single annular pMUT. The results show that both resonant frequency and velocity amplitude from the EQC models for individual transducers are in good agreement with the FEM simulations.

In the coupled CAC-pMUT, the phase of the driving voltage can be varied because the top electrodes of the curved and annular structures are independent. Both in-phase and anti-phase driving are available. The average velocity of the coupled CAC-pMUT can also be calculated using Equation (27), but the matrix of mechanical impedance and acoustic impedance needs to be modified as follows:

(30) $$Z_{mech}=diag\left\{Z_{m_{cur}},Z_{m_{sec}},\;\cdots ,\;Z_{m_{sec}}\right\}$$

(31) $${Z_{s}}_{\left(N+1\right)\times \left(N+1\right)}=Z_{self}+Z_{mutual}\;$$

(32) $$Z_{self}=diag\left\{\frac{Z_{a_{cur}}}{S_{cur}^{2}},\frac{{Z_{a}}_{sec}}{S_{sec}^{2}},\;\cdots ,\;\frac{{Z_{a}}_{sec}}{S_{sec}^{2}}\right\}$$

(33) $$Z_{mutual}=\left[\begin{array}{ccccc} 0 & \frac{Z_{12}}{S_{cur}S_{sec}} & \frac{Z_{13}}{S_{cur}S_{sec}} & \cdots & \frac{Z_{1\left(N+1\right)}}{S_{cur}S_{sec}}\\ \frac{Z_{21}}{S_{sec}S_{cur}} & 0 & \frac{Z_{23}}{S_{sec}^{2}} & \cdots & \frac{Z_{2\left(N+1\right)}}{S_{sec}^{2}}\\ \frac{Z_{31}}{S_{sec}S_{cur}} & \frac{Z_{32}}{S_{sec}^{2}} & 0 & \; & \vdots \\ \vdots & \vdots & \; & \ddots & \frac{Z_{N\left(N+1\right)}}{S_{sec}^{2}}\\ \frac{Z_{\left(N+1\right)1}}{S_{sec}S_{cur}} & \frac{Z_{\left(N+1\right)2}}{S_{sec}^{2}} & \cdots & \frac{Z_{\left(N+1\right)N}}{S_{sec}^{2}} & 0 \end{array}\right]\;$$

**Figure 6:**
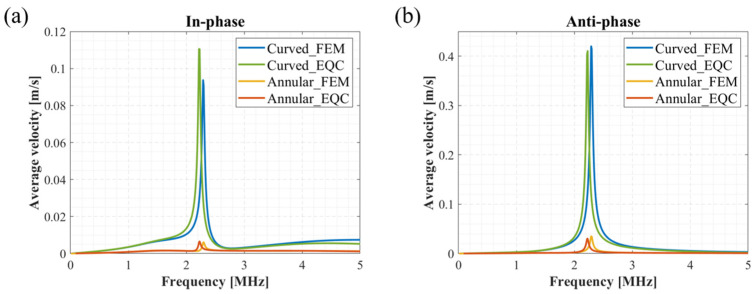
Comparison of FEM model results and EQC model results (**a**) under in-phase excitation and (**b**) under anti-phase excitation.

Based on the above impedance equations and Equation (27), the vibration velocities of the curved and annular pMUT can be separately calculated. The results are shown in Figure 6. For in-phase driving, a voltage of +1 V in amplitude is applied to the curved and annular pMUTs. For anti-phase driving, the curved structure is driven by a voltage of +1 V in amplitude, while the annular electrode is actuated by −1 V in amplitude. The results of the EQC model are in line with the FEM simulations, and the average velocities of the entire coupling structure is dramatically increased in the anti-phase driving compared to the in-phase excitation.

## 3. Results: Acoustic Field Analysis

As a first step towards the pressure analysis of the coupled CAC-pMUT, the in-water pressure calculation of the curved pMUT and the annular pMUT is demonstrated individually.

**Figure 7:**
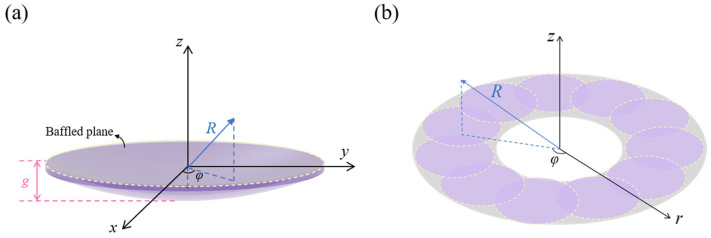
(**a**) Schematic of pressure field calculation for a curved pMUT. (**b**) Schematic of sound pressure calculation for an annular pMUT based on equivalent pistons.

Based on the theory of moving transducers in a flat rigid baffle (Figure 7a), the spatial acoustic pressure generated of a curved pMUT can be computed by the Rayleigh integral [12,37]:

(34) $$p_{cur}=\frac{j\rho_{m}ck}{2\pi }\iint \frac{u\left(r^{\prime }\right)}{R}e^{-jkR}r^{\prime }dr^{\prime }d\varphi$$

where *R* is the distance from a point on the transducer to a point in space, and *u*(*r*′) is the velocity at the baffled plane.

For the annular pMUT, the output pressure can be obtained by superimposing the pressure of its segmented circular pistons, as shown in Figure 7b. Considering that the dimension of each piston is much smaller than the ultrasound wavelength, the pressure at any arbitrary point can be approximated as a point source:

(35) $$p_{ann}=\sum_{n=1}^{N}\frac{j\rho_{m}ckv_{seci}}{2\pi }dir\left(\theta ,\varphi \right)\frac{e^{-jkR\left(n\right)}}{R\left(n\right)}$$

where *dir*(*θ*,*φ*) is the directivity function [37]:

(36) $$dir\left(\theta ,\varphi \right)=\frac{2J_{1}\left(kasin\theta \right)}{kasin\theta }$$

where *k* is the wave number, and *a* is the radius of the circular piston. When *ka* < 1, the value of *dir*(*θ*,φ) can be assumed to be 1.

When the curved pMUT and the annular pMUT are coupled together, the sound pressure at any spatial point is a superposition of the sound pressures of the two structures. Using the coupled EQC model, we calculate the acoustic pressure distribution of a single curved pMUT (rcur = 60 μm), a single annular pMUT (rann1 = 95 μm, wann = 135 μm), and the coupled two structures as CAC-pMUT with in-phase-driven and anti-phase-driven. All derived results are validated by the corresponding FEM simulations using COMSOL, while the vibration profiles at resonance frequencies are further examined using FEM.

**Figure 8:**
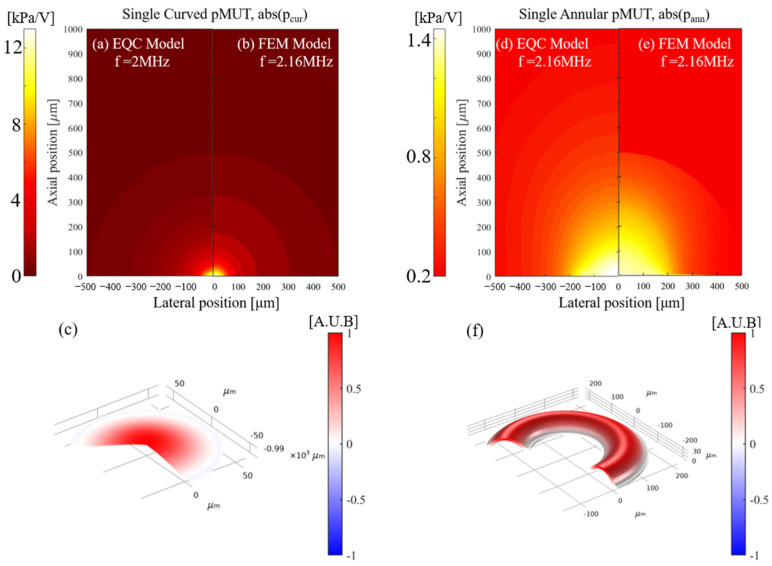
Schematic of 2D sound pressure field distribution and 3D vibration mode of a single curved pMUT (**a**–**c**) and a single annular pMUT (**d**–**f**). (**a**,**d**) Pressure results from EQC model. (**b**,**e**) Pressure results and (**c**,**f**) vibration mode from FEM model.

**Figure 9:**
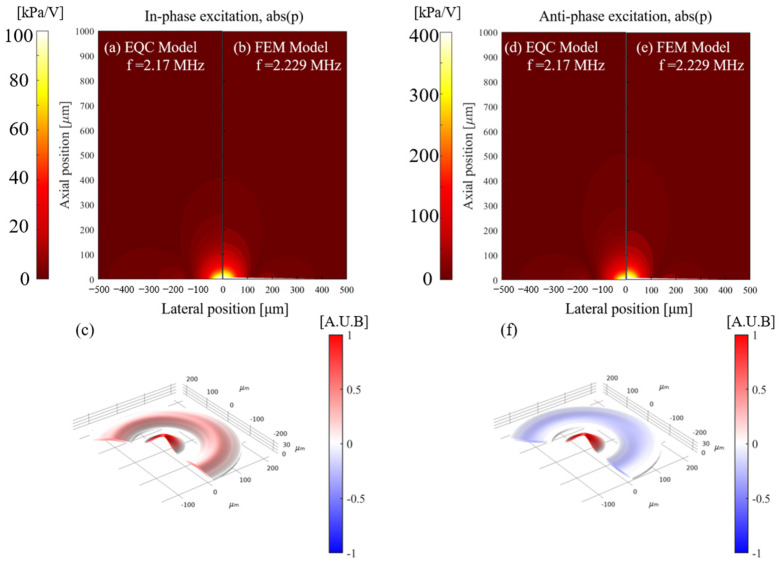
Schematic of 2D sound pressure field distribution and 3D vibration mode of an in-phase excitation CAC-pMUT (**a**–**c**) and an anti-phase excitation CAC-pMUT (**d**–**f**). (**a**,**d**) Pressure results from EQC model. (**b**,**e**) Pressure results and (**c**,**f**) vibration mode from FEM model.

In Figure 8, the single curved structure can provide an acoustic pressure 9 times higher than the single annual counterpart (12.7 kPa/V vs. 1.4 kPa/V), albeit working at the similar resonant frequency of around 2.1 MHz. After the annular and curved structures are concentrically coupled, CAC-pMUT with in-phase driving at the resonance frequency shows the same vibration direction of the two counterparts (Figure 9c), while the anti-phase driving one gives the opposite vibration directions (Figure 9f). Figure 9a,b have demonstrated that CAC-pMUT with in-phase driving can achieve pressure transmit efficiency as 100 kPa/V, higher than any of the single structure (i.e., transmit efficiency of single curved diaphragm 12.7 kPa/V, transmit efficiency of single annular diaphragm 1.4 kPa/V). It is worth mentioning that the acoustic pressure can be further maximized as 400 kPa/V by anti-phase driving (Figure 9d,e). As such, the coupled CAC-pMUT with anti-phase excitation can provide a sound pressure around 285 times higher compared with a single annular pMUT of the same size, significantly improving the ultrasound penetration depth and the acoustic SNC. There is a small pressure discrepancy in the side lobes between EQC and FEM in Figure 9a,b and Figure 9d,e. It is attributed to approximating the curved structure as a projected flat diaphragm in the coupled EQC, introducing the constant scalar *k_c_*_1_ as shown in Equation (15). However, the assumption still holds true due to the fact that the proposed curvature of the spherical vibration surface remains small (*R_cur_* ≫ *r_cur_*) and the calculated pressure in the primary direction or region aligns well between EQC and FEM. In summary, the overall pressure performance of the proposed EQC model has shown good agreement with the FEM simulations.

## 4. Discussion: Structure Optimization of the CAC-pMUT

Based on the EQC model of CAC-pMUTs, we investigate their structure and driving approach to optimize their output transmit efficiency. Firstly, the overall resonant frequency is fixed around 2 MHz due to the aimed applications, i.e., deep-tissue imaging, non-destructive testing, and particle manipulation. For that reason, we have to determine the size of the coupling device to obtain the target resonant frequency in the optimization. Firstly, the dimensions of the coupled curved and annular diaphragms are interrelated; specifically, their piezoelectric layers must share the same thickness, and the gap between the annular part and the curved part of the coupling structure has to be larger than 35 μm to ensure the etching quality of SOI [17]. Secondly, the curved diaphragm contributes dominantly to the coupled transmission efficiency. In light of these considerations, we fix the dimension of the curved device as a first step by sweeping the radius of curvature, the nominal radius, and the layer thickness. Subsequently we study the parameters of the annular structure, including the thickness of the piezoelectric layer and size of the inner diameter.

**Figure 10:**
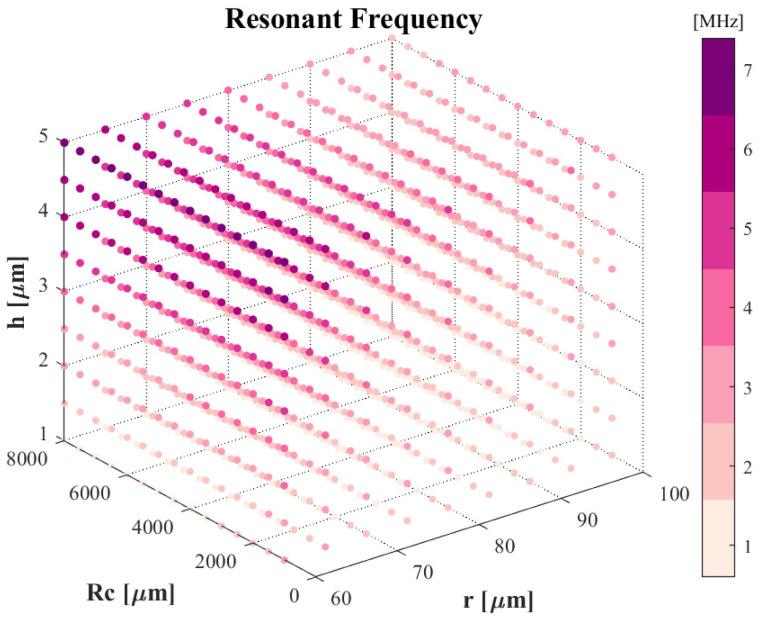
Frequency response matrix of a curved pMUT.

As shown in Figure 10, three parameters (radius of curvature *R_c_*, nominal radius *r*, and piezoelectric layer thickness *h*) can be optimized for a frequency around 2 MHz, initialized as 1000 μm for *R_c_*, 60 μm for *r*, and 3 μm for *h*. There are two constraints: (1) the radius of curvature must be high enough to keep the assumption that the spherical shape vibrates as the projected flat membrane, and (2) the piezoelectric layer’s thickness is limited due to that of the annular structure, necessitating an additional passive layer. Accordingly, the thickness of the piezoelectric layer in the annular part is also set as 3 μm and the inner radius is 95 μm. There are three other parameters to be optimized in the whole coupling CAC-pMUT: (1) the phases of two driving voltages, (2) the width of the annular structure, and (3) the thickness of the passive layer in the annular part.

**Figure 11:**
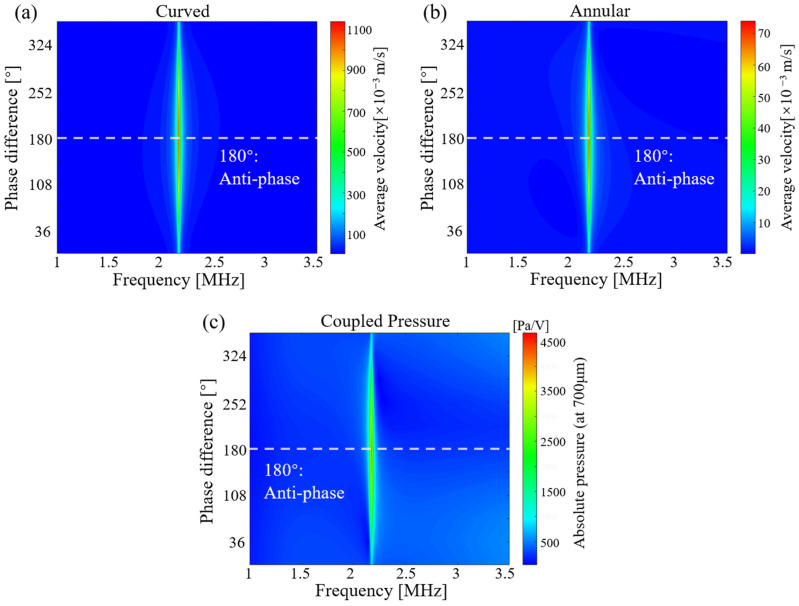
The effect on the results brought about by giving different phases to the voltages applied to the curved and annular structures after coupling. (**a**) Velocity–frequency response of the curved part. (**b**) Velocity–frequency response of the annular part. (**c**) Frequency response of the acoustic pressure at 700 μm for a coupled pMUT.

To investigate the impact of the phase difference, we firstly change the phase applied to the annular part from 0° to 360° while maintaining a constant amplitude for all driving voltages and keeping the phase of the voltage on the spherical part unchanged. As can be seen from Figure 11, when the phase difference is 180°, i.e., the CAC-pMUT in anti-phase driving, the average velocities of the annular and curved parts reach maximum at resonant frequency (approx. 2.3 MHz). Next, the change in sound pressure with phase difference at 700 μm from the coupling transducer is calculated, which also reaches the maximum at the anti-phase working mode. Based on the continuity equation of fluid, the average velocity of the vibrated diaphragms and the output pressure are maximized as 10 times higher at 180° phase difference due to the optimization of the acoustic interactions between the coaxial coupled structure. Therefore, anti-phase driving is selected for the subsequent analyses.

**Figure 12:**
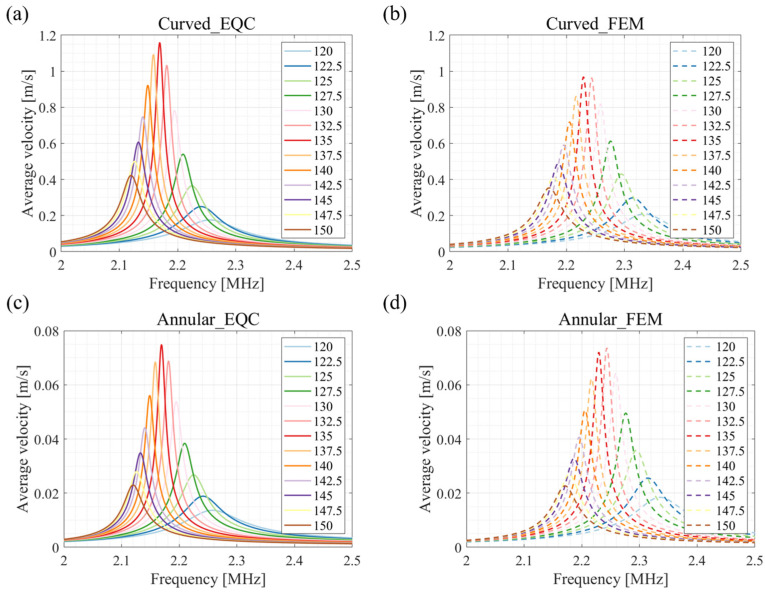
Effect of the width of the annular structure on transducer performance during anti-phase excitation. (**a**) Average velocity of curved part derived by EQC model. (**b**) Average velocity of curved part derived by FEM model. (**c**) Average velocity annular part derived by EQC model. (**d**) Average velocity of annular part derived by FEM model.

The influence of the annular width on the output average velocity is illustrated in Figure 12. From Figure 12a,b, it can be seen that the EQC derived vibration velocities of the annular diaphragms at different widths align well with the FEM references. Meanwhile, the output velocities of spherical structure from EQC are slightly higher than those of the finite element simulation due to the projection of the curved diaphragm into a flat diaphragm. However, all velocities reach a maximum at an annular width of 135 μm, both in coupled EQC analysis and in FEM simulations. As a result, an annular structure with a width of 135 μm is the option for generating maximum output performance in this study.

**Figure 13:**
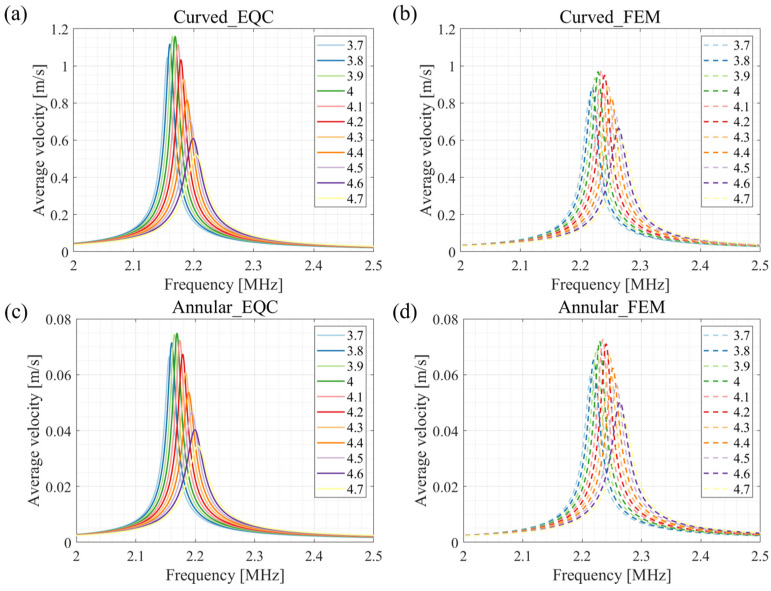
Effect of the thickness of the passive layer of the annular pMUT on transducer performance during anti-phase excitation. (**a**) Average velocity of curved part derived by EQC model. (**b**) Average velocity of curved part derived by FEM model. (**c**) Average velocity annular part derived by EQC model. (**d**) Average velocity of annular part derived by FEM model.

Finally, from 3.7 μm to 4.7 μm, the appropriate passive layer thickness of annular part is optimized using the EQC model. The effect of passive layer thickness on the average velocity of curved and annular parts agrees with the FEM simulation. As the thickness of the passive layer increases, the average velocity decreases. An amount of 4 μm is chosen as the thickness of the passive layer. In Figure 12 and Figure 13, there is a 2.5% frequency shift in EQC simulation compared to that of FEM simulation, probably because the FEM is conducted in multi-physics fields while EQC only considers interactions through the acoustic medium. In addition, there are discrepancies in the amplitude of average velocities between the EQC results and those from the FEM. We utilized the piston approximation method to determine the effective vibration area of the annular and curved membranes with equivalent volumetric displacement. In the optimization study, the effective vibration radius does not change accordingly with the varied dimensions, leading to the differences in the averaged velocities. However, the trends observed in both EQC and FEM results show good alignment, reaching a maximum at an annular width of 135 μm and a thickness of 4 μm.

**Figure 14:**
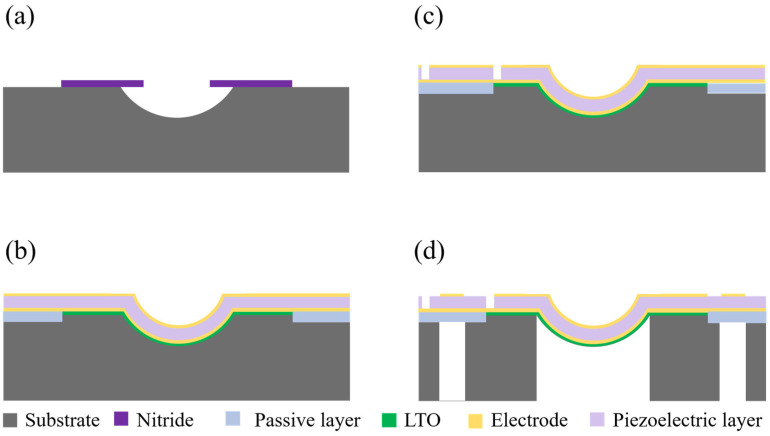
Process flow for the CAC-pMUT. (**a**) The curved structure substrate is formed using silicon wet etching with nitride as a mask. (**b**) A backside etching stop layer is formed using low pressure chemical vapor deposition (LPCVD) of low-temperature oxides (LTO), followed by deposition of a passive layer for the annular portion and sputtering of the bottom electrode/piezoelectric layer/top electrode stacking structure. (**c**) Via etching of the top electrode and piezoelectric layer in the curved and annular part, respectively, was performed using plasma etching to expose the bottom electrode. (**d**) A cavity is formed by backside deep reactive ion etching (DRIE) to release the diaphragm.

In Figure 5b, the single annular pMUT operating in a liquid medium shows a large bandwidth because the multiple resonance peaks merges. Coupling the annular and curved structures as CAC-pMUT can dramatically enhance the output performance but compress the bandwidth at the same time. As summarized in Table 3, the bandwidth results of equivalent circuit (EQC) models are consistent with the finite element (FEM) simulations, while bandwidth differences are small between the in-phase and the anti-phase excitation of coupled CAC-pMUTs. The increased transmit efficiency comes at the cost of a reduced bandwidth for the coupled CAC-pMUTs. Specifically, the bandwidth is decreased by a factor of 10 compared to a single curved pMUT and by approximately 50 compared to a single annular pMUT. However, the main purpose of the study is to enhance the transmit efficiency not the bandwidth, which is the dominant obstacle in the applications using continuous waves or modulated waves such as therapy [4,5], biomedical particle manipulation [6,7,8], and neural stimulation [10]. Moreover, we would like to address the possibility of enlarging the bandwidth for the CAC-pMUT array, which is important for medical imaging [1,2,3] and gesture recognition [9] applications. It could be achieved by placing cells of different sizes together in a fluid acoustic medium and utilizing the large damping effect of the acoustic medium to merge the multiple resonance peaks to achieve an increased bandwidth [15]. In addition, the fabrication of 3D curved structures is inherently challenging and needs further study to keep the polarization direction perpendicular to the surface through the whole piezoelectric layer [20]. We propose a CAC-PMUT manufacturing process, as shown in Figure 14. The curved structure substrate is first formed using silicon wet etching with nitride as a mask (Figure 14a). Then, a backside etching stop layer is formed using low pressure chemical vapor deposition (LPCVD) of low-temperature oxides (LTO), followed by deposition of a passive layer for the annular portion and sputtering of the bottom electrode/piezoelectric layer/top electrode stacking structure (Figure 14b). Via etching of the top electrode and piezoelectric layer in the curved and annular part, respectively, was performed using plasma etching to expose the bottom electrode (Figure 14c). Finally, a cavity is formed by backside deep reactive ion etching (DRIE) to release the diaphragm (Figure 14d).

In closely packed arrays, vibrating pMUT cells normally work in the same phase and thereafter amplify the acoustic interactions/crosstalk in between, which is considered a critical obstacle for pMUT arrays to realize desirable performance. For that reason, the proposed CAC-pMUT within one cell but driven in anti-phase is thus expected to improve the interactions, paving the way for high-performance large pMUT array with lower crosstalk. The EQC investigation of the CAC-pMUT array is ongoing.

## 5. Conclusions

This work proposes a new type of concentrically coupled pMUT structure CAC-pMUT containing curved and annular membranes, aiming to enhance the transmit efficiency for targeted applications such as deep-tissue imaging and neural stimulation. In order to fulfill the required operating frequency of 2 MHz, we initially establish the dimensional parameters of the curved structure, i.e., a radius of curvature of 1000 μm, a nominal radius of 60 μm, and a thickness of 3 μm. In order to characterize the key acoustic interactions, we develop a coupled EQC model of the curved pMUT and annular pMUT by incorporating mutual acoustic impedance. Utilizing the EQC model, we conduct a quantitative analysis to assess the impact of several factors on the overall output performance, including the width of the annular structure in the range of 120–150 μm, the thickness of the passive layer covering 3.7–4.7 μm, and the phase variation 0–360°of the excitation voltages. Validated by the FEM analysis, our study reveals that the coupled CAC-pMUT, optimized with an annulus width of 135 μm and a passive layer thickness of 4 μm, exhibits superior performance, particularly when actuated in anti-phase (with a phase difference of 180°). This configuration demonstrates an output sound pressure 285 times higher than that of a single-ring device of equivalent size. Furthermore, our EQC model offers computational efficiency over FEM simulations in designing CAC-pMUT arrays for future study, facilitating the quantitative characterization of dominant acoustic coupling phenomena across electrical, mechanical, and acoustical domains.

## Figures and Tables

**Table 1:** Geometry parameters of the CAC-pMUT.

Symbol	Description	Value
*R_cur_*	Radius of curvature	1000 μm
*r_cur_*	Nominal radius of curved pMUT	60 μm
*r_ann_*_1_	Inner radius of annular pMUT	95 μm
*r_ann_*_4_	Outer radius of annular pMUT	220 μm
*w_ann_*	Width of annular pMUT	*r_ann_*_4_–*r_ann_*_1_
*h*_1_	Thickness of Si	4 μm
*h*_2_	Thickness of AlN	3 μm

**Table 2:** Material parameters of the active and passive layer of the CAC-pMUT.

Symbol	Description	Value
*Y*_1_	Young’s modulus of Si	170 GPa
*v*_1_	Poisson’s ratio of Si	0.28
*ρ*_1_	Density of Si	2329 kg/m^3^
*Y*_2_	Young’s modulus of AlN	340 GPa
*v*_2_	Poisson’s ratio of AlN	0.3
*ρ*_2_	Density of AlN	3300 kg/m^3^
*d*_31_	Piezoelectric coefficient of AlN	2.2 pm/V

**Table 3:** Comparison of the bandwidth of the single curved pMUT, the single annular pMUT, the in-phase coupled CAC-pMUT, and the anti-phase coupled CAC-pMUT.

Bandwidth (−3 dB)	Single Curved	Single Annular	Coupled-In Phase		Coupled-Anti Phase	
			Curved	Annular	Curved	Annular
EQC	26.69%	181%	3.6%	4.04%	3.59%	3.59%
FEM	33%	184%	3.49%	3.91%	3.93%	3.93%

## Data Availability

The original contributions presented in the study are included in the article, further inquiries can be directed to the corresponding authors.
